# 17α-Ethynyl-3-methoxy­estra-1,3,5(10),9(11)-tetraen-17-ol

**DOI:** 10.1107/S1600536808005254

**Published:** 2008-04-02

**Authors:** Hongqi Li, Yanxi Song, Fengyan Ge

**Affiliations:** a College of Chemistry and Chemical Engineering, Donghua University, Shanghai 201620, People’s Republic of China; b College of Environmental Science and Engineering, Donghua University, Shanghai 201620, People’s Republic of China

## Abstract

In the title compound, C_21_H_24_O_2_, rings *B*, *C* and *D* adopt half-chair, distorted half-chair and envelope conformations, respectively. In the crystal structure, there is an inter­molecular O—H⋯O hydrogen bond. The mol­ecules are arranged in a head-to-tail fashion, with the meth­oxy and hydr­oxy groups forming a two-dimensional hydrogen-bond network.

## Related literature

For related literature, see: Doussot *et al.* (1995 ▶); Ekhato *et al.* (2002 ▶); Sedee *et al.* (1985 ▶); Steiner *et al.* (1997 ▶).
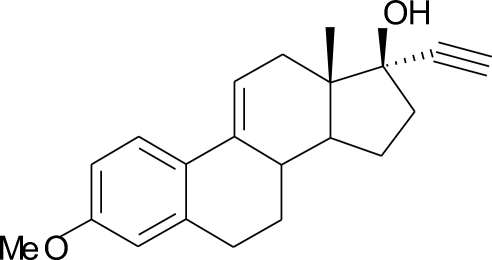


## Experimental

### 

#### Crystal data

C_21_H_24_O_2_
*M* *_r_* = 308.40
Orthorhombic, 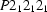

*a* = 7.3773 (6) Å
*b* = 10.7430 (9) Å
*c* = 21.2555 (18) Å
*V* = 1684.6 (2) Å^3^
*Z* = 4
Mo *K*α radiation
μ = 0.08 mm^−1^
*T* = 293 (2) K
0.50 × 0.43 × 0.34 mm

#### Data collection

Rigaku FCR CCD area-detector diffractometer
Absorption correction: multi-scan (*SADABS*; Sheldrick, 2004 ▶) *T*_min_ = 0.824, *T*_max_ = 1.000 (expected range = 0.802–0.974)
9960 measured reflections
2127 independent reflections
1884 reflections with *I* > 2σ(*I*)
*R*_int_ = 0.070

#### Refinement

*R*[*F* ^2^ > 2σ(*F* ^2^)] = 0.043
*wR*(*F* ^2^) = 0.114
*S* = 1.03
2127 reflections
214 parameters
H atoms treated by a mixture of independent and constrained refinement
Δρ_max_ = 0.20 e Å^−3^
Δρ_min_ = −0.24 e Å^−3^

### 

Data collection: *SMART* (Bruker, 2001 ▶); cell refinement: *SAINT* (Bruker, 2001 ▶); data reduction: *SAINT*; program(s) used to solve structure: *SHELXTL* (Sheldrick, 2008 ▶); program(s) used to refine structure: *SHELXTL*; molecular graphics: *SHELXTL*; software used to prepare material for publication: *SHELXTL*.

## Supplementary Material

Crystal structure: contains datablocks global, I. DOI: 10.1107/S1600536808005254/wn2237sup1.cif

Structure factors: contains datablocks I. DOI: 10.1107/S1600536808005254/wn2237Isup2.hkl

Additional supplementary materials:  crystallographic information; 3D view; checkCIF report

## Figures and Tables

**Table 1:** Hydrogen-bond geometry (Å, °)

*D*—H⋯*A*	*D*—H	H⋯*A*	*D*⋯*A*	*D*—H⋯*A*
O2—H3⋯O1^i^	0.82 (4)	2.14 (4)	2.933 (3)	163 (3)

Symmetry code: (i) 
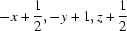
.

**Fig. 1.**
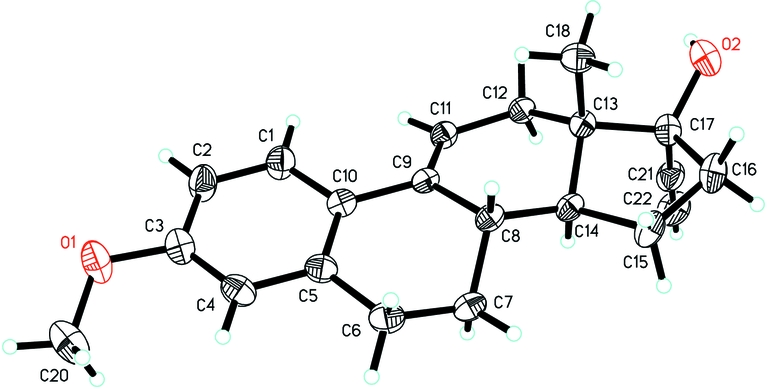
The molecular structure of the title compound. Displacement ellipsoids are drawn at the 30% probability level and H atoms are shown as small spheres of arbitrary radius.

**Fig. 2.**
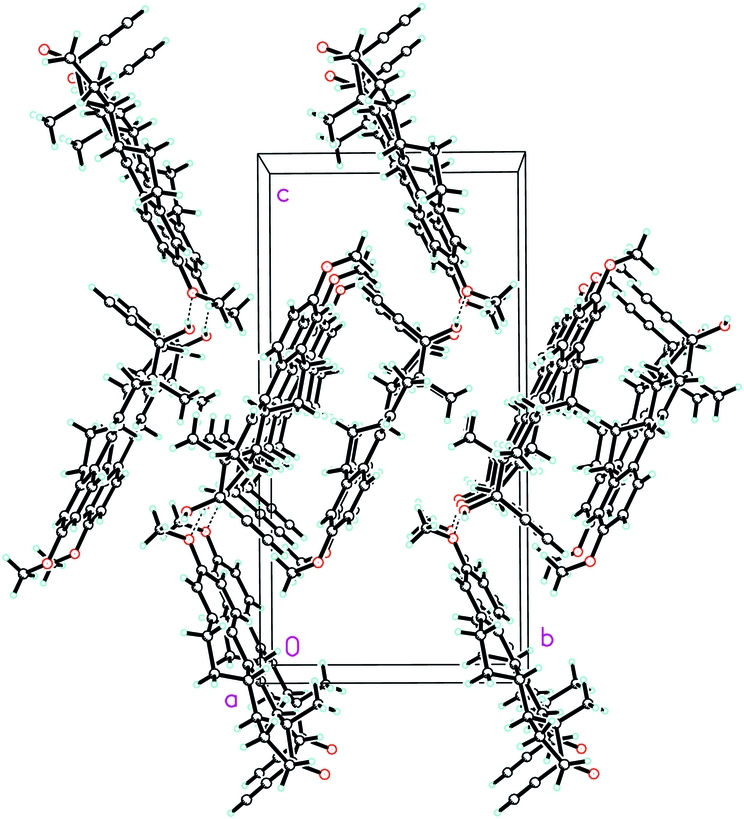
The crystal structure of the title compound, viewed along the a axis. Dashed lines indicate hydrogen bonds

## supplementary crystallographic information

### Comment

The title compound is a photodecomposition product of mestranol (Sedee *et al.*, 1985) and can be used as an intermediate for the synthesis of steroidal drugs. The preparation of the title compound starting from mestranol, through an oxidative dehydrogenation with 2,3-dichloro-5,6-dicyanoquinone (DDQ) in methanol, was reported by Doussot *et al.* (1995) However, no crystal structure of the title compound has been reported thus far. Here we present the crystal structure of 17α-ethinyl-3-methoxyestra-1,3,5(10),9(11)-tetraen-17-ol.

The geometry (Fig. 1) of the steroid skeleton does not differ significantly from that of mestranol (Steiner *et al.*, 1997), except, of course, for the C?C bond in ring C. There is an intermolecular hydrogen bond O2—H3···O1, but no intra- or intermolecular hydrogen bonding between hydroxy and ethynyl groups is observed. The molecules are arranged in a head-to-tail fashion, different from the head-to-head fashion observed in mestranol, with the methoxy and hydroxy groups forming a two-dimensional hydrogen bond network (Fig. 2).

### Experimental

The title compound was prepared, in 90% yield, by methylation of 3,17β-dihydroxy-19-norpregna-1,3,5(10),9(11)-tetraen-20-yne (Ekhato *et al.*, 2002) with methyl iodide and potassium carbonate at room temperature. Crystals suitable for X-ray diffraction were obtained by slow evaporation of a solution in ethyl acetate and petroleum ether (1:1, *v*/*v*).

### Refinement

The H atom bonded to O was located in a difference map and refined with a distance restraint of O—H = 0.82 (4) Å. Other H atoms were positioned geometrically and refined using a riding model, with C—H = 0.93 Å for benzene and acetylenic C—H, 0.96 Å for methyl C—H, 0.97 Å for methylene C—H, and 0.98Å for methine C—H; *U*_iso_(H) = 1.2*U*_eq_(C) except for methyl groups, where *U*_iso_(H) = 1.5*U*_eq_(C). In the absence of significant anomalous scattering effects, Friedel pairs were merged.

### Crystal data

**Table:**

C_21_H_24_O_2_	*D*_x_ = 1.216 Mg m^−^^3^
*M**_r_* = 308.40	Melting point: 145 K
Orthorhombic, *P*2_1_2_1_2_1_	Mo *K*α radiation λ = 0.71073 Å
*a* = 7.3773 (6) Å	Cell parameters from 3971 reflections
*b* = 10.7430 (9) Å	θ = 5.4–53.4º
*c* = 21.2555 (18) Å	µ = 0.08 mm^−^^1^
*V* = 1684.6 (2) Å^3^	*T* = 293 (2) K
*Z* = 4	Prismatic, colorless
*F*_000_ = 664	0.50 × 0.43 × 0.35 mm

### Data collection

**Table:**

Rigaku FCR CCD area-detector diffractometer	2127 independent reflections
Radiation source: fine-focus sealed tube	1884 reflections with *I* > 2σ(*I*)
Monochromator: graphite	*R*_int_ = 0.071
*T* = 293(2) K	θ_max_ = 27.0º
φ and ω scans	θ_min_ = 1.9º
Absorption correction: multi-scan(SADABS; Sheldrick, 2004)	*h* = −7→9
*T*_min_ = 0.824, *T*_max_ = 1.000	*k* = −13→9
9960 measured reflections	*l* = −25→27

### Refinement

**Table:**

Refinement on *F*^2^	Secondary atom site location: difference Fourier map
Least-squares matrix: full	Hydrogen site location: inferred from neighbouring sites
*R*[*F*^2^ > 2σ(*F*^2^)] = 0.044	H atoms treated by a mixture of independent and constrained refinement
*wR*(*F*^2^) = 0.114	*w* = 1/[σ^2^(*F*_o_^2^) + (0.0757*P*)^2^ + 0.0067*P*] where *P* = (*F*_o_^2^ + 2*F*_c_^2^)/3
*S* = 1.03	(Δ/σ)_max_ = 0.001
2127 reflections	Δρ_max_ = 0.20 e Å^−^^3^
214 parameters	Δρ_min_ = −0.24 e Å^−^^3^
Primary atom site location: structure-invariant direct methods	Extinction correction: none

### Special details

**Table:**

Geometry. All e.s.d.'s (except the e.s.d. in the dihedral angle between two l.s. planes) are estimated using the full covariance matrix. The cell e.s.d.'s are taken into account individually in the estimation of e.s.d.'s in distances, angles and torsion angles; correlations between e.s.d.'s in cell parameters are only used when they are defined by crystal symmetry. An approximate (isotropic) treatment of cell e.s.d.'s is used for estimating e.s.d.'s involving l.s. planes.
Refinement. Refinement of *F*^2^ against ALL reflections. The weighted *R*-factor *wR* and goodness of fit *S* are based on *F*^2^, conventional *R*-factors *R* are based on *F*, with *F* set to zero for negative *F*^2^. The threshold expression of *F*^2^ > σ(*F*^2^) is used only for calculating *R*-factors(gt) *etc*. and is not relevant to the choice of reflections for refinement. *R*-factors based on *F*^2^ are statistically about twice as large as those based on *F*, and *R*- factors based on ALL data will be even larger.

### Fractional atomic coordinates and isotropic or equivalent isotropic displacement parameters (Å^2^)

**Table:**

	*x*	*y*	*z*	*U*_iso_*/*U*_eq_	
O1	0.3222 (3)	0.77612 (18)	0.73106 (7)	0.0684 (5)	
O2	0.5307 (3)	0.26039 (16)	1.17027 (8)	0.0583 (5)	
C1	0.2555 (3)	0.6031 (2)	0.87459 (10)	0.0502 (5)	
H1	0.1618	0.5584	0.8932	0.060*	
C2	0.2245 (3)	0.6607 (2)	0.81810 (10)	0.0556 (6)	
H2	0.1108	0.6561	0.7994	0.067*	
C3	0.3626 (3)	0.7256 (2)	0.78916 (10)	0.0497 (5)	
C4	0.5296 (3)	0.7326 (2)	0.81737 (10)	0.0503 (5)	
H4	0.6227	0.7762	0.7978	0.060*	
C5	0.5612 (3)	0.67502 (19)	0.87522 (10)	0.0422 (5)	
C6	0.7485 (3)	0.6809 (2)	0.90286 (11)	0.0548 (6)	
H6A	0.7993	0.7628	0.8951	0.066*	
H6B	0.8250	0.6203	0.8820	0.066*	
C7	0.7495 (3)	0.6558 (2)	0.97282 (11)	0.0496 (5)	
H7A	0.6936	0.7251	0.9947	0.059*	
H7B	0.8737	0.6487	0.9873	0.059*	
C8	0.6478 (2)	0.53696 (19)	0.98837 (9)	0.0362 (4)	
H8	0.7043	0.4684	0.9652	0.043*	
C9	0.4518 (3)	0.54791 (17)	0.96698 (9)	0.0345 (4)	
C10	0.4221 (3)	0.60908 (18)	0.90521 (9)	0.0380 (4)	
C11	0.3154 (3)	0.50772 (19)	1.00285 (9)	0.0376 (4)	
H11	0.1985	0.5207	0.9878	0.045*	
C12	0.3331 (3)	0.44369 (19)	1.06504 (9)	0.0379 (4)	
H12A	0.2522	0.3726	1.0663	0.045*	
H12B	0.2980	0.5004	1.0984	0.045*	
C13	0.5267 (3)	0.40034 (17)	1.07587 (8)	0.0358 (4)	
C14	0.6545 (3)	0.50617 (18)	1.05775 (9)	0.0369 (4)	
H14	0.6133	0.5804	1.0804	0.044*	
C15	0.8389 (3)	0.4696 (2)	1.08594 (10)	0.0517 (6)	
H15A	0.9140	0.4288	1.0547	0.062*	
H15B	0.9023	0.5424	1.1015	0.062*	
C16	0.7939 (3)	0.3803 (3)	1.13995 (11)	0.0572 (6)	
H16A	0.8467	0.4097	1.1790	0.069*	
H16B	0.8408	0.2978	1.1311	0.069*	
C17	0.5855 (3)	0.3774 (2)	1.14476 (10)	0.0447 (5)	
C18	0.5615 (3)	0.28188 (19)	1.03758 (9)	0.0471 (5)	
H18A	0.5295	0.2962	0.9944	0.071*	
H18B	0.4893	0.2151	1.0541	0.071*	
H18C	0.6874	0.2601	1.0402	0.071*	
C20	0.4291 (5)	0.8770 (3)	0.70975 (14)	0.0782 (9)	
H19A	0.5512	0.8493	0.7030	0.117*	
H19B	0.4284	0.9419	0.7408	0.117*	
H19C	0.3801	0.9083	0.6710	0.117*	
C21	0.5210 (3)	0.4781 (2)	1.18698 (9)	0.0500 (5)	
C22	0.4707 (4)	0.5547 (3)	1.22112 (11)	0.0644 (7)	
H21	0.4303	0.6162	1.2485	0.077*	
H3	0.425 (5)	0.260 (3)	1.1812 (13)	0.079 (11)*	

### Atomic displacement parameters (Å^2^)

**Table:**

	*U*^11^	*U*^22^	*U*^33^	*U*^12^	*U*^13^	*U*^23^
O1	0.0730 (12)	0.0800 (12)	0.0523 (9)	0.0059 (10)	0.0010 (9)	0.0254 (8)
O2	0.0669 (13)	0.0551 (10)	0.0528 (9)	0.0104 (9)	0.0036 (8)	0.0111 (7)
C1	0.0378 (11)	0.0683 (14)	0.0445 (11)	−0.0046 (11)	0.0001 (9)	0.0071 (10)
C2	0.0426 (12)	0.0772 (16)	0.0472 (12)	0.0009 (12)	−0.0074 (9)	0.0103 (12)
C3	0.0527 (12)	0.0535 (12)	0.0429 (11)	0.0087 (11)	0.0024 (9)	0.0062 (10)
C4	0.0492 (13)	0.0485 (12)	0.0532 (12)	−0.0034 (10)	0.0119 (10)	0.0080 (10)
C5	0.0393 (11)	0.0396 (10)	0.0477 (11)	−0.0016 (8)	0.0050 (9)	−0.0006 (9)
C6	0.0379 (11)	0.0630 (14)	0.0634 (14)	−0.0149 (11)	0.0014 (10)	0.0112 (12)
C7	0.0365 (10)	0.0542 (12)	0.0580 (13)	−0.0110 (10)	−0.0068 (9)	0.0003 (10)
C8	0.0274 (8)	0.0414 (10)	0.0396 (10)	−0.0003 (8)	0.0002 (7)	−0.0059 (8)
C9	0.0311 (9)	0.0332 (9)	0.0392 (9)	−0.0010 (8)	−0.0021 (7)	−0.0051 (7)
C10	0.0350 (10)	0.0392 (10)	0.0399 (9)	0.0004 (8)	0.0028 (8)	−0.0037 (8)
C11	0.0276 (8)	0.0424 (10)	0.0429 (10)	0.0019 (8)	−0.0013 (8)	−0.0005 (8)
C12	0.0321 (10)	0.0410 (10)	0.0406 (10)	−0.0013 (8)	0.0003 (8)	−0.0013 (8)
C13	0.0342 (9)	0.0373 (9)	0.0358 (9)	0.0037 (8)	−0.0004 (7)	−0.0044 (7)
C14	0.0301 (9)	0.0410 (10)	0.0397 (9)	0.0024 (8)	−0.0031 (7)	−0.0086 (8)
C15	0.0348 (11)	0.0690 (15)	0.0515 (12)	0.0035 (11)	−0.0083 (9)	−0.0047 (11)
C16	0.0463 (12)	0.0728 (16)	0.0525 (13)	0.0130 (12)	−0.0124 (10)	0.0021 (11)
C17	0.0474 (11)	0.0477 (11)	0.0390 (10)	0.0075 (10)	−0.0031 (8)	0.0003 (9)
C18	0.0522 (12)	0.0395 (10)	0.0496 (11)	0.0024 (10)	0.0042 (10)	−0.0091 (9)
C20	0.101 (2)	0.0648 (16)	0.0685 (16)	0.0106 (18)	0.0222 (16)	0.0199 (13)
C21	0.0557 (13)	0.0572 (13)	0.0369 (10)	−0.0028 (11)	−0.0062 (9)	−0.0045 (10)
C22	0.0740 (17)	0.0709 (16)	0.0482 (12)	0.0020 (15)	−0.0018 (12)	−0.0185 (12)

### Geometric parameters (Å, °)

**Table:**

O1—C3	1.381 (3)	C11—C12	1.496 (3)
O1—C20	1.415 (4)	C11—H11	0.9300
O2—C17	1.428 (3)	C12—C13	1.520 (3)
O2—H3	0.82 (4)	C12—H12A	0.9700
C1—C2	1.370 (3)	C12—H12B	0.9700
C1—C10	1.392 (3)	C13—C14	1.526 (3)
C1—H1	0.9300	C13—C18	1.532 (3)
C2—C3	1.379 (3)	C13—C17	1.547 (3)
C2—H2	0.9300	C14—C15	1.538 (3)
C3—C4	1.373 (3)	C14—H14	0.9800
C4—C5	1.396 (3)	C15—C16	1.532 (3)
C4—H4	0.9300	C15—H15A	0.9700
C5—C10	1.400 (3)	C15—H15B	0.9700
C5—C6	1.503 (3)	C16—C17	1.542 (3)
C6—C7	1.511 (3)	C16—H16A	0.9700
C6—H6A	0.9700	C16—H16B	0.9700
C6—H6B	0.9700	C17—C21	1.483 (3)
C7—C8	1.517 (3)	C18—H18A	0.9600
C7—H7A	0.9700	C18—H18B	0.9600
C7—H7B	0.9700	C18—H18C	0.9600
C8—C14	1.512 (3)	C20—H19A	0.9600
C8—C9	1.520 (2)	C20—H19B	0.9600
C8—H8	0.9800	C20—H19C	0.9600
C9—C11	1.334 (3)	C21—C22	1.159 (3)
C9—C10	1.484 (3)	C22—H21	0.9300
			
C3—O1—C20	117.8 (2)	C11—C12—H12B	109.5
C17—O2—H3	113 (2)	C13—C12—H12B	109.5
C2—C1—C10	122.4 (2)	H12A—C12—H12B	108.0
C2—C1—H1	118.8	C12—C13—C14	108.29 (15)
C10—C1—H1	118.8	C12—C13—C18	109.35 (17)
C1—C2—C3	119.7 (2)	C14—C13—C18	112.42 (16)
C1—C2—H2	120.1	C12—C13—C17	117.09 (16)
C3—C2—H2	120.1	C14—C13—C17	100.62 (15)
C4—C3—C2	119.7 (2)	C18—C13—C17	108.90 (16)
C4—C3—O1	124.2 (2)	C8—C14—C13	112.88 (15)
C2—C3—O1	116.0 (2)	C8—C14—C15	117.70 (17)
C3—C4—C5	120.7 (2)	C13—C14—C15	104.93 (16)
C3—C4—H4	119.7	C8—C14—H14	106.9
C5—C4—H4	119.7	C13—C14—H14	106.9
C4—C5—C10	120.2 (2)	C15—C14—H14	106.9
C4—C5—C6	118.6 (2)	C16—C15—C14	105.10 (17)
C10—C5—C6	121.13 (19)	C16—C15—H15A	110.7
C5—C6—C7	112.43 (18)	C14—C15—H15A	110.7
C5—C6—H6A	109.1	C16—C15—H15B	110.7
C7—C6—H6A	109.1	C14—C15—H15B	110.7
C5—C6—H6B	109.1	H15A—C15—H15B	108.8
C7—C6—H6B	109.1	C15—C16—C17	106.14 (18)
H6A—C6—H6B	107.9	C15—C16—H16A	110.5
C6—C7—C8	111.26 (18)	C17—C16—H16A	110.5
C6—C7—H7A	109.4	C15—C16—H16B	110.5
C8—C7—H7A	109.4	C17—C16—H16B	110.5
C6—C7—H7B	109.4	H16A—C16—H16B	108.7
C8—C7—H7B	109.4	O2—C17—C21	108.75 (17)
H7A—C7—H7B	108.0	O2—C17—C16	109.0 (2)
C14—C8—C7	112.36 (16)	C21—C17—C16	110.2 (2)
C14—C8—C9	109.86 (15)	O2—C17—C13	114.86 (18)
C7—C8—C9	109.89 (17)	C21—C17—C13	111.54 (17)
C14—C8—H8	108.2	C16—C17—C13	102.34 (18)
C7—C8—H8	108.2	C13—C18—H18A	109.5
C9—C8—H8	108.2	C13—C18—H18B	109.5
C11—C9—C10	122.52 (17)	H18A—C18—H18B	109.5
C11—C9—C8	121.42 (17)	C13—C18—H18C	109.5
C10—C9—C8	116.03 (16)	H18A—C18—H18C	109.5
C1—C10—C5	117.21 (18)	H18B—C18—H18C	109.5
C1—C10—C9	121.55 (18)	O1—C20—H19A	109.5
C5—C10—C9	121.24 (17)	O1—C20—H19B	109.5
C9—C11—C12	126.00 (18)	H19A—C20—H19B	109.5
C9—C11—H11	117.0	O1—C20—H19C	109.5
C12—C11—H11	117.0	H19A—C20—H19C	109.5
C11—C12—C13	110.91 (15)	H19B—C20—H19C	109.5
C11—C12—H12A	109.5	C22—C21—C17	178.4 (2)
C13—C12—H12A	109.5	C21—C22—H21	180.0
			
C10—C1—C2—C3	1.2 (4)	C11—C12—C13—C14	45.3 (2)
C1—C2—C3—C4	−0.4 (4)	C11—C12—C13—C18	−77.5 (2)
C1—C2—C3—O1	177.2 (2)	C11—C12—C13—C17	158.11 (17)
C20—O1—C3—C4	−24.5 (3)	C7—C8—C14—C13	170.07 (16)
C20—O1—C3—C2	158.0 (2)	C9—C8—C14—C13	47.4 (2)
C2—C3—C4—C5	−0.2 (4)	C7—C8—C14—C15	−67.4 (2)
O1—C3—C4—C5	−177.6 (2)	C9—C8—C14—C15	169.92 (17)
C3—C4—C5—C10	0.0 (3)	C12—C13—C14—C8	−65.2 (2)
C3—C4—C5—C6	177.5 (2)	C18—C13—C14—C8	55.8 (2)
C4—C5—C6—C7	160.3 (2)	C17—C13—C14—C8	171.47 (15)
C10—C5—C6—C7	−22.3 (3)	C12—C13—C14—C15	165.44 (16)
C5—C6—C7—C8	50.9 (3)	C18—C13—C14—C15	−73.6 (2)
C6—C7—C8—C14	177.20 (18)	C17—C13—C14—C15	42.07 (18)
C6—C7—C8—C9	−60.2 (2)	C8—C14—C15—C16	−149.88 (18)
C14—C8—C9—C11	−13.5 (3)	C13—C14—C15—C16	−23.4 (2)
C7—C8—C9—C11	−137.59 (19)	C14—C15—C16—C17	−4.8 (2)
C14—C8—C9—C10	164.70 (15)	C15—C16—C17—O2	152.64 (18)
C7—C8—C9—C10	40.6 (2)	C15—C16—C17—C21	−88.1 (2)
C2—C1—C10—C5	−1.3 (3)	C15—C16—C17—C13	30.6 (2)
C2—C1—C10—C9	178.8 (2)	C12—C13—C17—O2	80.7 (2)
C4—C5—C10—C1	0.7 (3)	C14—C13—C17—O2	−162.21 (17)
C6—C5—C10—C1	−176.7 (2)	C18—C13—C17—O2	−43.9 (2)
C4—C5—C10—C9	−179.47 (17)	C12—C13—C17—C21	−43.6 (3)
C6—C5—C10—C9	3.1 (3)	C14—C13—C17—C21	73.5 (2)
C11—C9—C10—C1	−14.8 (3)	C18—C13—C17—C21	−168.21 (19)
C8—C9—C10—C1	167.05 (19)	C12—C13—C17—C16	−161.36 (18)
C11—C9—C10—C5	165.4 (2)	C14—C13—C17—C16	−44.3 (2)
C8—C9—C10—C5	−12.8 (3)	C18—C13—C17—C16	74.0 (2)
C10—C9—C11—C12	179.06 (17)	O2—C17—C21—C22	25 (10)
C8—C9—C11—C12	−2.9 (3)	C16—C17—C21—C22	−95 (10)
C9—C11—C12—C13	−14.1 (3)	C13—C17—C21—C22	152 (10)

### Hydrogen-bond geometry (Å, °)

**Table:**

*D*—H···*A*	*D*—H	H···*A*	*D*···*A*	*D*—H···*A*
O2—H3···O1^i^	0.82 (4)	2.14 (4)	2.933 (3)	163 (3)

Symmetry codes: (i) −*x*+1/2, −*y*+1, *z*+1/2.
